# Research on the relationship between work-family conflict and burnout among civil aviation pilots after the COVID-19 pandemic

**DOI:** 10.1371/journal.pone.0340375

**Published:** 2026-01-02

**Authors:** Weiheng Chen, Shouxi Zhu, Kequan Shao, Kejia Liang

**Affiliations:** Flight College, Shandong University of Aeronautics, Binzhou, China; All India Institute of Medical Sciences, INDIA

## Abstract

To examine the impact of work-family conflict on occupational burnout among pilots following the COVID-19 pandemic, this study employed the Maslach Burnout Inventory-General Survey and the Zhao Xinyuan Bi-directional Scale of Work-Family Conflict as research instruments. The data were analyzed using correlation analysis and Structural Equation Modeling (SEM) to explore the relationship between work-family conflict and burnout. The results indicate a significant correlation between work-family conflict and burnout, as well as the three dimensions of burnout. Specifically, the correlation coefficients between work interfering with family (WIF) and family interfering with work (FIW) with occupational burnout were found to be 0.737 and 0.496, respectively. In the path analysis of the SEM, the estimated effects of WIF and FIW on burnout were both 0.49. Mediation analysis revealed that WIF indirectly influences burnout through FIW, with a mediation effect value of 0.117. Additionally, while pilot-related factors did not significantly affect burnout, they were found to have a notable impact on work-family conflict. These findings underscore the significant role of work-family conflict in contributing to burnout and provide a theoretical foundation for targeted interventions aimed at mitigating burnout among pilots.

## 1 Introduction

Since the end of the COVID-19 outbreak, the global economy has exhibited a gradual recovery, with the aviation industry demonstrating a pattern of steady resumption similar to its pre-outbreak levels. According to forecasts by the International Air Transport Association (IATA), all regional carriers are projected to achieve consecutive years of profit growth by 2024, with the Asia-Pacific region experiencing the most substantial growth [1]. Given the significant global presence of air transport, with approximately 4.1 billion passengers travelling on civil airliners each year [2], the role of pilots as direct aircraft operators to ensure flight safety is paramount. Technical competence, decision-making skills, and psychological well-being are critical factors in maintaining flight safety.

For aviation practitioners, high-intensity work pressure, irregular working hours, and the responsibility of handling emergency situations can lead to anxiety, depression, or sleep disorders [3]. Some individuals rely on medication to regulate their condition [4]. Additionally, the nature of the profession can also result in social isolation and strained family relationships.Demerouti et al. found that 40% of commercial airline pilots had very high burnout symptoms [5]. Pilots working in a long-term, high-pressure, high-intensity work environment will gradually develop burnout, which can lead to feelings of emotional, mental, and physical exhaustion, as well as a sense of alienation from the profession [6]. Burnout has been shown to have a detrimental effect on pilots’ performance in the execution of flight tasks, and in serious cases, it can hurt flight safety [7]. The underlying causes of pilot burnout are multifaceted, including, but not limited to, the demanding nature of the work environment, protracted flight missions, and irregular work and rest schedules. Other contributing factors include emotional volatility, stagnant career progression, and inadequate family support. These factors often stem from a confluence of interconnected issues [8].

The COVID-19 pandemic has had a profound impact on pilots’ mental health, with significant emotional stress undermining their psychological and social well-being [9]. Studies have shown that short-term isolation during the COVID-19 period did not have a significant effect on pilots’ mental health; however, when the isolation period extended to 9–10 weeks or longer, 28.3% of pilots experienced moderate depression, and 12.5% exhibited severe depression [10][Karien, 2022 #234][Karien, 2022 #234]. Another survey revealed that more than one-third of pilots displayed symptoms of diagnosable mental health disorders during the pandemic [11]. Xu et al. [12] found that proactive coping mechanisms, by reducing perceived stress, contributed to the improvement of pilots’ mental health, providing theoretical foundations and practical recommendations for mental health interventions in the aviation industry under the ongoing pandemic control measures.

Following the conclusion of the epidemic, pilots were transitioned back to their regular work status from their home state. The extended period spent in their homes may have resulted in a shift in their work rhythms and lifestyle habits. The need to readjust to the high-stress, high-responsibility flight environment may induce psychological distress, and the high-intensity work state may lead to the accumulation of fatigue in pilots, affecting their judgment and decision-making abilities and triggering burnout. Burnout can manifest as dissatisfaction with the job and a sense of alienation from the profession [13]. Pilot burnout, characterized by a state of exhaustion and disengagement in the workplace, has been demonstrated to manifest in a decline in concentration and alertness during flight, leading to an increased propensity for errors [14]. This decline in vigilance can be further exacerbated by mood swings and fatigue, which have been shown to directly impact the safety of flight operations and efficacy of emergency response procedures.

Work-family conflict is a prevalent issue across various professions, particularly in occupations that require remote work or frequent travel [15]. Professions such as seafarers and pilots, due to the unique nature of their work, often necessitate prolonged absences from home, which limits their participation in daily family activities and caregiving. This imbalance is conducive to work-family conflict, which can adversely affect individuals’ emotional needs, health, and work performance [16,17].Given that work and family represent a significant proportion of a pilot’s time and energy, it is theoretically significant to study pilot burnout from the perspective of work and family [18].

In the latter half of the 20th century, social life underwent a marked acceleration in pace, with the balance between work and family becoming a significant factor in the harmony of life. Kopelman et al. [19] advanced the theory of work-family role conflict, formulating a role conflict model that conceptualizes individual role conflict as a consequence of stressful incompatibility between roles. Subsequent scholars have expanded on this theory by incorporating gender, socio-cultural, and policy factors into their research and analyzing it at different levels: individual, organizational, and policy [20,21], and further categorizing work-family conflict into work interfering with family and family interfering with work [22].

Work-family conflict refers to the contradiction that arises when the demands of work and family roles are difficult to reconcile, usually manifested as work interfering with family or family interfering with work. This conflict can have various negative impacts on individuals, families, work, and society [23]. The work-family conflict is influenced by numerous factors and has a detrimental effect on individual human well-being and behavior [24]. However, life satisfaction can, to a certain extent, enhance job satisfaction, and work-family conflict exerts a moderating influence on this relationship [25]. Furthermore, work-family balance has a mediating effect on the relationship between work-family conflict and job satisfaction [26]. To achieve a state of equilibrium between professional obligations and personal commitments, corporate entities are advised to formulate suitable policies that facilitate the coordination of work-family obligations [27]. The implementation of such policies has been shown to mitigate adverse emotional responses and promote optimal mental well-being [28]. In the context of the airline industry, the presence of organizational support and the influence of collective culture within airlines have been demonstrated to exert an effect on work-family conflict [29]. Work-family conflict has been shown to increase psychological tension in workers [30], and for pilots, stress from family has been linked to work fatigue, which in turn affects behavior at work [31].

Burnout is used to describe extreme psychological or emotional fatigue that occurs when a worker is overworked [32]. It has been associated with various factors, including work environment, excessive workload, interpersonal tension, and role ambiguity [33]. To quantify burnout, Maslach et al. [34] classified it into three dimensions: emotional exhaustion, dehumanization, and low personal accomplishment, and developed a burnout measurement questionnaire. The development of this scale has spanned five decades since its introduction, with numerous scholars researching burnout among workers in various fields. These studies have consistently demonstrated that burnout has a detrimental effect on worker performance [35].

In recent years, burnout has emerged as a salient issue for aviation practitioners such as pilots and controllers [36]. While the complete elimination of stress in their work is an unattainable goal, there is a need to investigate coping mechanisms to effectively manage it [37]. The formation of pilot burnout is multifaceted, with individual, organizational, and environmental factors contributing to its development to varying extents [33]. The imbalance between give and take at work has been identified as a potential trigger for burnout, while factors such as social support and job satisfaction have been shown to have a mitigating effect [38,39].

A review of the extant literature reveals the prevalence of research on both work-family conflict and burnout in numerous fields. Research on work-family conflict has primarily focused on three aspects: individual differences, organizational factors, and psycho-emotional effects. Conversely, burnout research focuses on workload, work pressure, work environment, and individual characteristics. The intertwined nature of work-family conflict and burnout in an individual’s working life is evident, with the two factors jointly affecting an individual’s psychological health and work performance. However, research on the combination of these two is limited. Work-family conflict and burnout may exhibit slight variations among individuals over time. The most recent research on both topics was conducted during the COVID-19 pandemic. However, there is a paucity of research on individuals post-epidemic, particularly in industries that were severely impacted, such as the airline industry, where research on both burnout and work-family conflict is a priority.

Prior to further research, work-family conflict was categorized into two distinct categories, namely work interfering with family (WIF) and family interfering with work (FIW), as proposed by Frone et al. [22]. The following hypotheses were formulated:

Hypothesis 1 (H1): There is a significant effect of WIF on pilot burnout.
Hypothesis 2 (H2): FIW has a significant effect on pilot burnout.
Hypothesis 3 (H3): Pilots’ own factors have a significant effect on their burnout.
Hypothesis 4 (H4): Pilots’ own factors have a significant effect on their WIF.
Hypothesis 5 (H5): There is a significant effect of pilots’ own factors on their FIW.
Hypothesis 6 (H6): There is a significant effect between WIF and FIW.

## 2 Method

### 2.1 Participants

Between November 10 and 13, 2024, this study distributed an online questionnaire to active pilots from Shandong Airlines. A total of 311 questionnaires were collected, with 306 valid responses; the questionnaire completion rate was 100%. To ensure the representativeness and appropriateness of the sample, and to guarantee the participation of various groups, such as different job positions and age stages, the collection of participants’ basic information was necessary to ensure the authenticity and scientific validity of the data obtained. The participant holds a valid pilot license, has not been subject to extended suspension or permanent reassignment from flight duties within the past 12 months due to medical or operational performance issues, and has not recently experienced significant psychological trauma, major surgery, or life-altering events that could impact operational performance or mental health.

Due to the specificity of the pilot’s profession, after excluding five invalid questionnaires, the majority of the sample was male, and there was only one female, of whom 75% were co-pilots. The largest number of subjects were in the below 30 and 30–40 age groups, with most having flown less than 1,000 hours. The majority of pilots possessed a bachelor’s degree, were not married, and did not hold an administrative position. Table 1 details the basic information of the pilots who participated in the survey.

**Table 1:** Basic information of participants. (N = 306).

Factors	Type	Frequency	Percentage
Gender	Male	305	99.67
	Female	1	0.33
Rank of pilot	Instructor	38	12.42
	Copilot	230	75.16
	Captain Pilot	38	12.42
Age group	Under 30 years old	132	43.14
	30-40	151	49.35
	41-50	19	6.21
	Over 50 years old	4	1.31
Educational background	Junior college or below	3	0.98
	Bachelor’s degree	300	98.04
	Master’s degree or higher	3	0.98
Total flight time	Within 1000 hours	119	38.89
	1000-3000	55	17.97
	3000-5000	54	17.65
	5000-10000	39	12.75
	More than 10000 hours	39	12.75
Marital status	Married	94	30.72
	Single	212	69.28
Administrative position	Yes	8	2.61
	No	298	97.39

This study was conducted in accordance with the Declaration of Helsinki and approved by the Ethics Committee of Shandong University of Aviation. All participants were adults, and the research process and data will remain anonymous, ensuring that participants’ privacy is protected. The experimental design and protocol of this study fully consider the principles of safety and fairness, ensuring that no harm is caused to the participants. If any participant disagreed, they were automatically exited from the survey. As the questionnaire was completed online, we only obtained verbal consent from the participants.

### 2.2 Survey questionnaire design

Current questionnaires for work-family conflict and burnout are relatively mature, and in this study, we used the Maslach Burnout Inventory Generic Survey [34] and the Zhao Xinyuan Bi-directional Scale of Work-Family Conflict [23] to survey the pilots.

The MBI is the most widely used burnout measurement tool, and is divided into three dimensions: emotional exhaustion, cynicism, and reduced personal accomplishment, with a total of 13 questions. The Work–Family Conflict Questionnaire has two dimensions, with a total of 10 questions: Work Interfering with Family (WIF), with 5 questions, and Family Interfering with Work (FIW), with 5 questions, where the 10th question is reverse scored.

To improve the reliability and validity of the statistical scales, the questionnaires were all scored on Likert seven point scale, which is naturally more sensitive to respondents, ranging from 1 to 7 (strongly disagree to strongly agree).

After collecting the questionnaires, the data were statistically analyzed using Excel 2021, Amos (version 26) and SPSS (version 25) data analysis platforms, and the data were examined using statistical methods such as correlation analysis.

### 2.3 Reliability and validity analysis

Although the questionnaire used was more mature, the reliability and validity of the questionnaire were tested to make the scale more reliable, and the subsequent study was more convincing. As shown in Tables 2 and 3, Cronbach’s *α* of the Burnout Questionnaire and its three dimensions and the Work-Family Conflict Questionnaire and its two dimensions are all greater than 0.7, which indicates good reliability; the data reliability is high, and there is a high degree of internal consistency between the dimensions of the scale, which can be tested in the next step. Using KMO and Bartlett’s test for validity verification, from Table 2, we can see that the burnout questionnaire and work-family conflict questionnaire KMO value is greater than 0.8; Bartlett’s spherical test probability of significance *P* < 0.001, there is a significant correlation between the variables, the data obtained from the questionnaire is reliable, very suitable for extracting information, and can be analyzed according to the study.

**Table 2:** Reliability testing.

	Dimensions	*n*	Cronbach’s *α*
Burnout	Emotional Exhaustion	4	0.752
	Cynicism	4	
	Reduced Personal Accomplishment	5	
Work-Family Conflict	WIF	5	0.880
	FIW	5	

**Table 3:** Validity testing.

	Dimensions	*n*	KMO	Approximate chi-square	DF	*P*
Burnout	Emotional Exhaustion	4	0.924	3610.402	105	0.000
	Cynicism	4				
	Reduced Personal Accomplishment	5				
Work-Family Conflict	WIF	5	0.885	2713.368	45	0.000
	FIW	5				

## 3 Results

### 3.1 Correlation analysis

The correlation between pilot burnout, WIF and FIW was analyzed using SPSS, and the results are presented in Table 4. The analysis revealed significant correlations between all pairs of variables. Among the three dimensions of burnout, emotional exhaustion was found to be significantly positively correlated with cynicism, while reduced personal accomplishment was significantly negatively correlated with both emotional exhaustion and cynicism. The overall level of burnout exhibited a positive correlation with emotional exhaustion and cynicism, while demonstrating a negative correlation with reduced personal accomplishment.

**Table 4:** Correlation between variables.

Variables	Emotional exhaustion	Cynicism	Reduced personal accomplishment	Burnout	WIF	FIW
Emotional exhaustion	1					
Cynicism	0.774**	1				
Reduced personal accomplishment	−0.468**	−0.612**	1			
Burnout	0.930**	0.853**	−0.281**	1		
WIF	0.744**	0.632**	−0.314**	0.737**	1	
FIW	0.516**	0.599**	−0.498**	0.496**	0.500**	1

* *P *< 0.05 ** *P *< 0.01.

Between burnout and work-family conflict, emotional exhaustion and cynicism were significantly positively correlated with both WIF and FIW. Conversely, reduced personal accomplishment demonstrated a significant negative correlation with both WIF and FIW. The findings indicate that pilots experiencing more pronounced work-family conflict tend to exhibit higher levels of burnout.

Significant correlations were observed between the variables, providing support for some of the hypotheses. These findings suggest a general direction for confirmatory factor analysis and the construction of structural equation models, paving the way for subsequent research.

### 3.2 Confirmatory factor analysis

**Fig 1:**
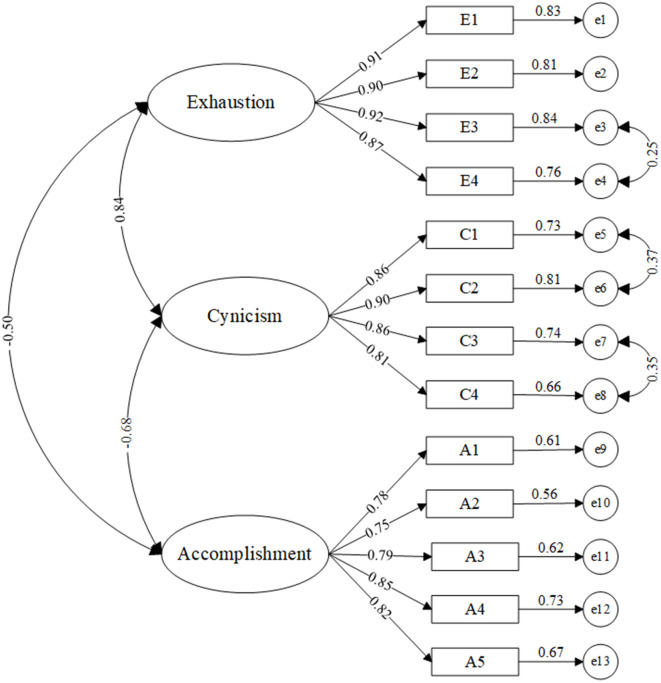
CFA model of pilots burnout.

As illustrated in Fig 1, the three dimensions of occupational burnout are conceptualized as latent variables, with multiple items within each dimension serving as observed variables. Confirmatory Factor Analysis (CFA) was conducted using Amos (Version 26) by constructing a measurement model and applying the Maximum Likelihood Estimation method. To ascertain the validity of the model, this study adopted the following parameters for the assessment of fit: a CMIN/DF value of less than 3.00, an RMSEA of less than 0.080, and values for GFI, CFI, and NFI of greater than 0.900 [40].

In the initial model construction, certain indicators did not meet the required criteria, with CMIN/DF and RMSEA failing to reach the acceptable thresholds. This indicated the need for model modification. Through the analysis of the questionnaire items and the consideration of the modification indices, bidirectional correlations were identified between the measurement errors e3 and e4, e5 and e6, as well as e7 and e8. This approach led to a reduction in the degrees of freedom by adding paths, thereby significantly improving the model fit for the pilot burnout model. Consequently, the model evaluation indicators attained acceptable ranges.

The fitting results of the revised model are shown in Table 5. The CMIN/DF value is 2.682, which is less than 3.00; the RMSEA is 0.074, which is less than 0.080, 90% CI [0.060, 0.088]; and the GFI, CFI, NFI and TLI values are all greater than 0.90. On the basis of the aforementioned data, it can be concluded that the overall fit of the measurement model meets theoretical expectations. In conclusion, the model fit is satisfactory, and the constructed model can be accepted.

**Table 5:** Goodness of fit indices for pilots burnout.

Index	CMIN/DF	CFI	GFI	NFI	TLI	RMSEA
Before correction	3.667	0.954	0.895	0.938	0.942	0.094
After correction	2.682	0.972	0.930	0.957	0.963	0.074

**Fig 2:**
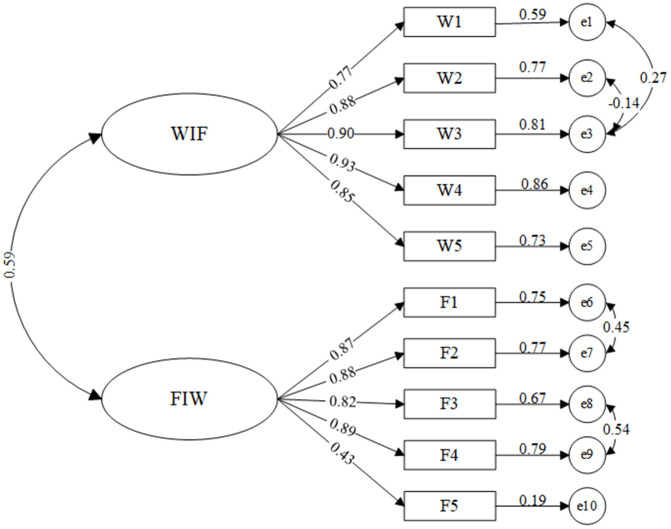
CFA model of work-family conflict.

Similarly, the model was constructed with WIF and FIW as latent variables and several items under each dimension as observed variables. Fig 2 shows the confirmatory factor analysis model for work-family conflict after modification. By establishing bidirectional correlations between the measurement errors e1 and e2, e1 and e3, e6 and e7, as well as e8 and e9, the model’s fit indices were improved. The fit indices of the revised model are shown in Table 6. The CMIN/DF value is 2.068, which is less than 3.00; RMSEA is 0.059, which is less than 0.080, 90% CI [0.038, 0.080]; and the GFI, CFI, NFI and TLI values are all greater than 0.90. Therefore, the constructed model can be accepted based on the fit indices.

**Table 6:** Goodness of fit indices for work-family conflict.

Index	CMIN/DF	CFI	GFI	NFI	TLI	RMSEA
Before correction	6.141	0.935	0.875	0.924	0.914	0.130
After correction	2.068	0.988	0.962	0.977	0.982	0.059

The CR value is indicative of the consistency with which all items in each latent variable explain that latent variable. A CR value should exceed 0.6. The AVE value is indicative of the average explanatory power of a latent variable over its observed variables, and the AVE value should be greater than 0.5 [41]. As shown in Table 7, the CR values of all variables meet the standard, indicating that the reliability and consistency of the questionnaire are considered acceptable. Additionally, the AVE values are all greater than 0.5, demonstrating the strong convergent validity of the questionnaire. Furthermore, Table 7 also lists the standardized factor loadings, unstandardized factor loadings, standard errors, t-values, and significance levels for burnout and work-family conflict. These results are significant at the 0.01 level, indicating that the measurement model exhibits good validity and reliability.

**Table 7:** Estimate of all the factors.

Variate	Item	Std. Estimate	CR	AVE	Unstandardized			
					Estimate	S.E.	t-value	*P*
Exhaustion	E1	0.913	0.944	0.810	1.000	–	–	–
	E2	0.898			1.028	0.041	24.898	***
	E3	0.917			0.993	0.038	26.009	***
	E4	0.870			1.005	0.044	22.604	***
Cynicism	C1	0.858	0.918	0.737	1.000	–	–	–
	C2	0.899			1.047	0.040	26.499	***
	C3	0.861			0.839	0.045	18.455	***
	C4	0.813			0.870	0.052	16.815	***
Accomplishment	A1	0.778	0.897	0.636	1.000	–	–	–
	A2	0.747			0.892	0.066	13.609	***
	A3	0.789			0.846	0.058	14.535	***
	A4	0.854			1.043	0.065	15.961	***
	A5	0.817			0.913	0.060	15.149	***
WIF	W1	0.766	0.937	0.749	1.000	–	–	–
	W2	0.876			1.138	0.069	16.564	***
	W3	0.899			1.197	0.061	19.757	***
	W4	0.926			1.174	0.066	17.724	***
	W5	0.852			1.077	0.067	16.095	***
FIW	F1	0.869	0.833	0.635	1.000	–	–	–
	F2	0.875			1.015	0.038	26.892	***
	F3	0.821			0.954	0.068	14.087	***
	F4	0.890			0.958	0.062	15.500	***
	F5	0.434			0.540	0.072	7.540	***

* *P* < 0.05, ** *P* < 0.01, *** *P* < 0.001.

### 3.3 Structural equation modeling analysis

**Fig 3:**
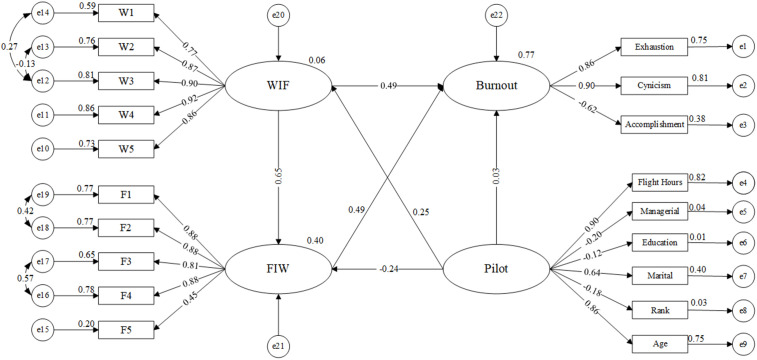
The finalized SEM model.

Structural equation modeling (SEM) is a multivariate statistical analysis technique that can simultaneously address complex relationships among multiple variables, incorporating both direct and indirect effects. It is especially suited for studying scenarios where various factors interact and has been widely applied in psychology and social science research. Building on the confirmatory factor analysis (CFA) of burnout and work-family conflict while adding more factors related to the pilots themselves, an SEM was developed. The final SEM, illustrated in Fig 3, incorporates the relationships among the pilot-related factors (Pilot), work-family conflict (WIF and FIW), and burnout. This SEM enables a comprehensive understanding of how these factors interact and influence one another, providing insights into the key pathways contributing to pilot burnout and work-family conflict.

As illustrated in Table 8, the relevant fit indices for the structural equation model are presented. The CMIN/DF value is 2.508, which is less than 3.00, and the RMSEA is 0.070, which is less than 0.080, 90% CI [0.061, 0.079]. Except for the GFI value of 0.888, which is marginally less than 0.90, the CFI, NFI and TLI values are both greater than 0.90. These results suggest that the model fits well overall and can be accepted as an adequate representation of the relationships between the variables.

**Table 8:** Model fit results.

Index	CMIN/DF	CFI	GFI	NFI	TLI	RMSEA
Value	2.508	0.947	0.888	0.916	0.937	0.070

The standardized estimation parameters between observed and latent variables in the model indicate the degree of correlation between them. The path coefficients of latent variables reflect the extent of their mutual influence. The results in Table 9 confirm that hypotheses H1, H2, H4, H5, and H6 are supported. Specifically, WIF and FIW have a significant positive impact on pilot burnout. This finding further emphasizes the critical role of work-family conflict in contributing to pilot burnout.

**Table 9:** Results for hypotheses verification.

Hypotheses	Path	Std. Estimate	S.E.	t-value	*P*	Result
H1	WIF→Burnout	0.489	0.049	8.502	***	Supported
H2	FIW→Burnout	0.487	0.168	6.136	***	Supported
H3	Pilot→Burnout	0.034	0.095	0.807	0.42	Unsupported
H4	Pilot→WIF	0.245	0.163	3.966	***	Supported
H5	Pilot→FIW	−0.237	0.065	−3.849	***	Supported
H6	WIF → FIW	0.649	0.038	6.791	***	Supported

* *P* < 0.05, ** *P* < 0.01, *** *P* < 0.001.

Hypothesis H3 is not supported, indicating that in this study model, the pilots’ factors do not have a direct significant impact on burnout. This could be due to the model not fully considering other mediating variables, or it may suggest that the influence of the pilots’ factors on burnout operates through other indirect pathways.

### 3.4 Mediating effect test

The results of the mediation effect test using the Bootstrap method, as shown in Table 10, indicate that FIW partially mediates the relationship between WIF and burnout. Specifically, the total effect of WIF on burnout is 1.015, with a 95% confidence interval of [0.911, 1.120] and a *P*-value less than 0.01, suggesting that the overall effect of WIF on burnout is significant. The direct effect of WIF on burnout is 0.898, with a 95% confidence interval of [0.780, 1.0160] and a *P*-value less than 0.01, indicating that the direct effect is also significant. The indirect effect of WIF on burnout through FIW is 0.117, with a 95% confidence interval of [0.031, 0.144] and a *P*-value less than 0.01. Since the confidence interval does not include 0, the mediation effect is significant. This suggests that WIF influences burnout partly through its direct effect and partly through FIW as a mediator. The effect of WIF on FIW is 0.257, and the effect of FIW on burnout is 0.456, with both *P*-values less than 0.01. Since both the direct and indirect effects are positive, this indicates that the mediation effect is partial. In conclusion, WIF has a direct effect on burnout, and part of its influence is mediated by FIW, demonstrating a partial mediation.

**Table 10:** Mediating effect test.

Parameter	Effect	95% CI		*P*
		Lower	Upper	
(Indirect) WIF→FIW→Burnout	0.117	0.031	0.144	**
WIF→FIW	0.257	0.207	0.307	**
FIW→Burnout	0.456	0.226	0.685	**
(Direct) WIF→Burnout	0.898	0.780	1.016	**
(Total) WIF→Burnout	1.015	0.911	1.120	**

* *P* < 0.05, ** *P* < 0.01.

WIF directly affects pilots’ occupational burnout, while also amplifying their sense of burnout through FIW. As pilots, they are required to maintain high levels of mental focus and work efficiency. However, due to the accumulation of negative emotions at home, they begin to feel inner unrest and anxiety, which makes it difficult to concentrate during work. Prolonged stress and burnout gradually accumulate, ultimately leading to feelings of burnout and physical fatigue during work.

## 4 Discussion

The ongoing global pandemic has had an unprecedented impact on the aviation industry, with pilots facing numerous challenges [42], including contract modifications and reduced income, which have had a significant impact on their mental health and cognitive functioning [43]. In this context, the relationship between work-family conflict and burnout is a complex and interacting issue. The nature of pilots’ work demands extended periods of time away from home, frequently entailing irregular work schedules and elevated levels of stress during flight duties [44]. These factors impact not only their occupational status, but also their family life, creating challenges for pilots and their loved ones. The crux of work-family conflict lies in the challenge pilots face in achieving equilibrium between professional obligations and family responsibilities, an imbalance that can further exacerbate their burnout.

In relevance research, WIF and FIW, as two dimensions of work-family conflict, are related to emotional exhaustion, cynicism, low sense of achievement, and career burnout. These relationships reflect the impact of the interaction between work and family life on an individual’s psychological and career development. WIF and FIW are positively correlated with emotional exhaustion, cynicism, and career burnout, indicating that conflicts between work and family intensify psychological stress, reducing individual work motivation and satisfaction [45]. Simultaneously, they are negatively correlated with a low sense of achievement, suggesting that conflicts between family and work affect an individual’s sense of self-worth and accomplishment, and may even lead to feelings of professional failure.

Pilot burnout is not caused by a single, isolated factor; individual, organizational, and societal factors all influence pilots and collectively contribute to emotional fatigue [46], lack of motivation, and the decline in work performance associated with burnout. Taking airlines as an example, airlines can effectively reduce pilot burnout and improve job satisfaction and safety by optimizing the work environment, providing reasonable working hours and task allocation, enhancing career support for employees, increasing management transparency, and offering competitive compensation and benefits. When constructing the structural equation model, it is challenging, on the one hand, to include all relevant factors as latent variables, and on the other hand, we only considered the impact of work-family conflict on burnout, without accounting for the work-family conflict arising from burnout. Therefore, the model we developed has certain limitations.

Of the more than 300 pilots surveyed, only one was female, which may have imposed some limitations on the results. The aviation industry’s unique characteristics have resulted in a relatively low proportion of female pilots. The most recent global survey on the gender status of pilots, published by the International Civil Aviation Organization (ICAO), revealed that the global proportion of female pilots was 4.69%, with the Asia-Pacific region exhibiting a slightly higher proportion of 6.15% [47]. The subsequent phase of the study could involve expanding the scope to include a larger number of participants and an increased proportion of female pilots, which may yield more robust conclusions.

## 5 Conclusion

The present study explores the relationship between pilots’ burnout and work-family conflict. To this end, four latent variables have been selected, and a model established on the correlations between these variables. A total of 19 observed variables were selected for the study, and the model was constructed using CFA for burnout and work-family conflict, followed by SEM. The model’s primary focus is on the impact of work-family conflict on pilot burnout. Empirical research was conducted to test the hypotheses proposed, thereby clarifying the path relationships between various influencing factors. The findings indicate that both WIF and FIW have a significant impact on burnout, with WIF exerting an indirect influence on burnout through the mediating effect of FIW. The findings further demonstrate that while pilots’ factors exert a substantial influence on WIF and FIW, their direct impact on occupational burnout was not found to be statistically significant. The findings of this study suggest that to alleviate pilots’ burnout, efforts should be concentrated on the reduction of WIF and FIW. Furthermore, it is recommended that attention be directed towards pilots’ factors to mitigate their impact on work-family conflict.

Pilots should strive to maintain a balance between their work and family life, ensuring open communication with their families, organizing family time effectively, and seeking the understanding and support of family members. Rest periods are of paramount importance for pilots, as a well-structured leave schedule can aid in the recovery of their energy and alleviate the stress associated with their demanding work. Airlines, in consideration of pilots’ work schedules and family needs, should provide adequate rest and vacation time. Additionally, airlines and relevant regulatory bodies should place greater emphasis on the psychological well-being of pilots by offering mental health services, providing psychological counseling and stress management training, and regularly assessing pilots’ professional burnout levels to offer timely adjustments and support. These measures can assist pilots in achieving a balance between work-family conflict and occupational burnout, enabling them to better care for their families and personal health, while also ensuring the long-term development of their professional careers.
